# Further investigation of blockade effects and binding affinities of selected natural compounds to immune checkpoint PD-1/PD-L1

**DOI:** 10.3389/fonc.2022.995461

**Published:** 2022-09-12

**Authors:** Huifang Li, Navindra P. Seeram, Chang Liu, Hang Ma

**Affiliations:** Bioactive Botanical Research Laboratory, Department of Biomedical and Pharmaceutical Sciences, College of Pharmacy, University of Rhode Island, Kingston, Rhode Island, United States

**Keywords:** immune checkpoint, PD-1/PD-L1, natural products, pair ELISA, surface plasmon resonance, binding affinity

## Abstract

The breakthrough in the discovery of immune checkpoint PD-1/PD-L1 inhibitors, such as the series of Bristol Myers Squibb synthetic compounds, boosted the research of small molecules with blockade effects on the interaction of PD-1/PD-L1. However, the search for natural products derived PD-1/PD-L1 inhibitors can be impeded by the false positive and/or negative results from the screening assays. Herein, we combined a PD-1/PD-L1 blockade assay (pair ELISA) and a PD-L1/PD-L1 binding assay (surface plasmon resonance; SPR) to evaluate a panel of natural compounds previously reported to show anti-PD-1/PD-L1 activity. The test compounds included kaempferol, cosmosiin, tannic acid, pentagalloyl glucose, ellagic acid, resveratrol, urolithin A, and rifubutin. Based on the analyses of their responses to the combined screening assays, these compounds were categorized into four groups: I) PD-1/PD-L1 inhibitors that can bind to PD-1 and PD-L1; II) PD-1/PD-L1 inhibitors selectively bind to PD-L1 protein; III) PD-1/PD-L1 inhibitors without binding capacity, and IV) PD-1/PD-L1 binders without blockade effect. Discrimination of positive responders in the PD-1/PD-L1 blockade and binding assays can provide useful insights to avoid false outcomes. Examples demonstrated in this study suggest that it is crucial to adopt proper evaluation methods (including using multiple-facet functional assays and target binding techniques) for the search for natural products derived PD-1/PD-L1 inhibitors.

## Introduction

Programmed cell death protein 1 (PD-1), a cell surface receptor expressed by T and B cells, is an immune checkpoint that regulates the immune system (1). PD-1 interacts with its ligands, including the programmed cell death-ligand 1 (PD-L1) expressed by antigen-presenting cells or cancer cells, to maintain immune homeostasis. In the tumor microenvironment, when PD-L1 binds to PD-1, T cell’s immune responses are undermined, which impairs its function of recognizing cancer cells. Consequently, cancer cells escaping from the immunological surveillance of T cells can lead to the proliferation of cancer cells and the expansion of tumor tissues (2). Thus, disrupting the interaction between PD-1 and PD-L1 has become a promising strategy for cancer immunotherapy. Molecules (e.g. immune checkpoint inhibitors) with blockade effects on the interaction of PD-1/PD-L1 can block the immune escaping signals of cancer cells and, consequently, reactivate immune cells to restore their anti-tumor response. Several antibody-based PD-1 inhibitors (e.g. Pidilizumab and Nivolumab) and PD-L1 inhibitors (e.g. Atezolizumab and Durvalumab) have been approved by the U.S. Food and Drug Administration (FDA) for the treatment of various cancers (3). Although these antibody drugs have shown potent anti-cancer efficacy in clinical trials, they are facing several inherent limitations, such as toxic off-target effects, poor permeability (of the tumor tissues), and immunogenicity, as well as prohibitive costs and challenging quality control (4, 5). On the contrary, compared to antibody drugs, small molecule derived PD-1/PD-L1 inhibitors may display a more favorable safety profile (6). Several synthetic small molecules with potent blockade effects on PD-1/PD-L1 have been developed. For instance, compounds including BMS1166 and BMS202 (by Bristol Myers Squibb Pharma Co.) are designed to directly bind the PD-L1 protein and consequently blockade the interaction of PD-1/PD-L1 (7). The strategy of combining both functional assay (for PD-1/PD-L1 inhibition) and binding characterization (for binding affinity to PD-1/PD-L1 proteins) to search for natural compounds including kaempferol (8), cosmosiin (9), resveratrol (10)and rifabutin (11) with anti-PD-1/PD-L1 effects has been adopted by our group and others. Our group has also summarized the methodologies for the development of small molecule based PD-1/PD-L1 inhibitors utilizing proper functional assays and biophysical methods (12). During the investigation, we noticed that several natural compounds (e.g. punicalagin) were identified as ‘false hits’ as they showed a detectable binding capacity to PD-1/PD-L1 proteins (characterized by surface plasmon resonance; SPR) but were not active in the PD-1/PD-L1 blockade assay (assessed by pair ELISA). This discrepancy is crucial for screening small molecules based PD-1/PD-L1 inhibitors given that the blockade effect of small molecules and their binding capacity to PD-1/PD-L1 proteins should be scrutinized to avoid false positives. Herein, in this current study, a panel of natural compounds with previously reported anti-PD-1/PD-L1 activity were selected to study the relationship between their blockade effects on PD-1/PD-L1 interaction (by pair ELISA assay) and binding capacity to the PD-1/PD-L1 proteins (by SPR assay). In addition, a series of natural polyphenols including tannic acid, pentagalloyl glucose, and urolithin A, which have similar chemical structure moieties as punicalagin, were included in the screening. Based on their positive responses to either the blockade assay or the binding affinity assay, these compounds were sorted in four categories: I) PD-1/PD-L1 inhibitors that bind to both PD-1 and PD-L1 proteins; II) PD-1/PD-L1 inhibitors that only bind to PD-L1 protein; III) PD-1/PD-L1 inhibitors without the binding capacity to PD-1/PD-L1 proteins; and IV) PD-1/PD-L1 binders with no blockade effect.

## Materials and methods

### Chemicals and reagents

BMS202 and BMS1166 were purchased from MedChemExpress LLC (Monmouth Junction, NJ, USA). Rifubutin, kaempferol, tannic acid, and cosmosiin were purchased from Cayman Chemical (Ann Arbor, MI, USA). Ellagic acid and resveratrol were purchased from Sigma Chemical Co. (St. Louis, MO, USA). Urolithin A (13) and pentagalloyl glucose (14) were synthesized and isolated, respectively, by our group as previously reported. Phosphate-buffered saline (PBS, pH 7.2) and dimethylsulfoxide (DMSO) were purchased from Thermo Fisher Scientific (Waltham, MA, USA). The test compounds were dissolved in DMSO (at 100 mM as a stock solution) and stored at -80°C for further use.

### PD-1/PD-L1 function assay with pair ELISA

The PD-l/PD-L1 blockade effect was determined by a pair ELISA assay kit (ACRO Biosystems, Newark, DE, USA). Briefly, human PD-L1 (200 ng/per well) was coated into a 96-well microplate and incubated overnight at 4°C. Next, the plate was washed with washing buffer followed by incubating with blocking buffer at room temperature for 1.5 h. Test samples were then added (at concentrations of 10 and 100 μM dissolved in dilution buffer with 0.1% of DMSO) prior to adding human PD-1-biotin (200 ng/per well). The plate was incubated at room temperature for 1 h. After horseradish peroxidase-conjugated streptavidin was added and incubated at room temperature for 1 h, the substrate was added into each well and incubated at room temperature for 20 min. The stop solution was then added to each well followed by measuring the absorbance of each well at a wavelength of 450 nm using a SpectraMax M2 plate reader (Molecular Devices; Sunnyvale, CA, USA).

### PD-1/PD-L1 binding assay with SPR

The PD-1/PD-L1 binding affinity of test natural compounds was measured on a SPR Biacore T200 instrument (GE Healthcare; Marlborough, MA, USA). Human PD-L1and PD-1 proteins (both with a Fc Tag) were purchased from ACRO Biosystems (Newark, DE, USA). Carboxymethylated CM 5 SPR chips were purchased from GE Healthcare (Marlborough, MA, USA). The SPR binding channels were set as: Cell-1, blank immobilization; Cell-2, human PD-L1 protein was immobilized by the injection of protein solution (40 μg/mL) in sodium acetate buffer (10 mM; pH 4.5); Cell-3, human PD-1 protein was immobilized by the injection of protein solution (40 μg/mL) in sodium acetate buffer (10 mM; pH 5.0). Approximately, 5500 and 4500 RU of human PD-1 and PD-L1 proteins, respectively, were immobilized on the flow cells with an amine coupling method.

## Results and discussion

### Category I: PD-1/PD-L1 inhibitors bind to PD-1 and PD-L1 (tannic acid and kaempferol)

**Figure 1:**
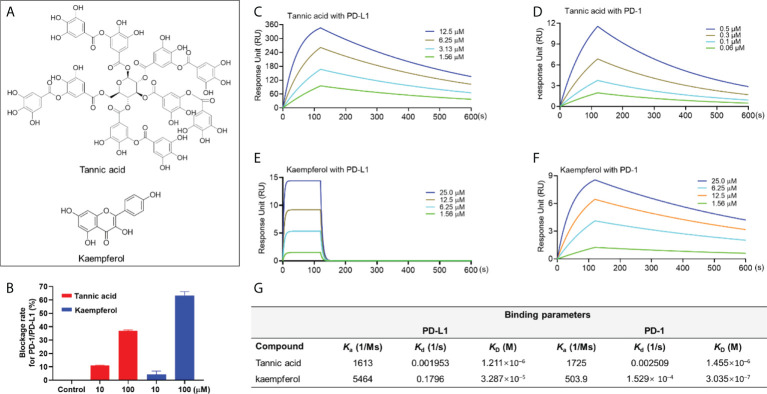
Chemical structure of TA and kaempferol **(A)**. Blockade rate of TA and kaempferol (at 10 and 100 μM) on the PD-1/PD-L1 interaction determined by the pair ELISA assay **(B)**. Binding profile of natural compounds at various concentrations with PD-1 and PD-L1 proteins characterized by the SPR measurements. Sensograms of TA with PD-L1 **(C)** or PD-1 **(D)** protein at 1.56-12.5 μM and 0.06-0.5 μM, respectively. Sensograms of kaempferol with PD-L1 **(E)** and PD-1 **(F)** protein at 1.56-25 μM. Summarized binding parameters including Ka, Kd, and K_D_ between test compounds and PD-L1 and PD-1 proteins **(G)**.

Natural compounds including tannic acid (TA) and kaempferol (**Figure 1A**) were identified as active PD-1/PD-L1 inhibitors by data from a combination of pair ELISA and the SPR binding assay. TA and kaempferol (10 and 100 μM) blockaded the PD-1/PD-L1 interaction by 11.0 and 36.9% and 4.1 and 63.4%, respectively (**Figure 1B**). TA and kaempferol also bound to PD-1 and PD-L1 proteins in the SPR measurements (**Figures 1C–F**). The binding capacity between TA and PD-1 or PD-L1 protein was identical with a comparable K_D_ value of 1.46×10^-6^ and 1.21×10^-6^ M, respectively. Kaempferol had a stronger binding affinity to PD-1 protein (K_D_ = 3.04 ×10^-7^ M) than PD-L1 protein (K_D_ = 3.3×10^-5^ M). The positive controls, BMS1166 and BMS202, showed a potent blockade effect (99.3 and 87.4% at 10 µM, respectively) and a strong binding affinity to PD-L1 (K_D_ = 5.7×10^-9^ M and 3.20 ×10^-7^ M, respectively). The positive results obtained in this category must be carefully examined. Although these compounds, in a manner similar to the positive controls, displayed both blockade effects and binding capacity to PD-1/PD-L1, TA and kaempferol had far less potent anti-PD1/PD-L1 effects as compared to BMS202. This is possibly due to BMS202’s distinct mechanism of blockading PD-1/PD-L1 interaction. BMS202 binds to PD-L1 and subsequentially induces the dimerization of PD-L1 monomers (15). The complex of dimerized PD-L1 and BMS202 further prevents the interaction with PD-1, which contributes to the dissociation of the PD-1/PD-L1 complex. This principle may not be applicable to the case of natural compounds (e.g. TA and kaempferol) due to the lack of data supporting that they can induce the dimerization of PD-L1, despite they can bind to PD-L1. Moreover, kaempferol’s binding affinity to PD-1 protein was 100-fold stronger than PD-L1 protein, whilst TA’s PD-1 and PD-L1 binding affinities were comparable. This observation suggests that TA and kaempferol did not selectively bind to PD-L1, and thus, may not be able to induce the dimerization of PD-L1 to exert the anti-PD-1/PD-L1 effect. Apart from the ambiguous mechanisms of action, the development of these natural compounds for PD-1/PD-L1 inhibitors can be challenging due to their low druggability (e.g. low bioavailability and non-specific targets) (16). Therefore, further studies using physiological relevant models to examine the effectiveness of compounds in this category are warranted to avoid false positives.

### Category II: PD-1/PD-L1 inhibitors selectively bind to PD-L1 protein (resveratrol and cosmosiin)

**Figure 2:**
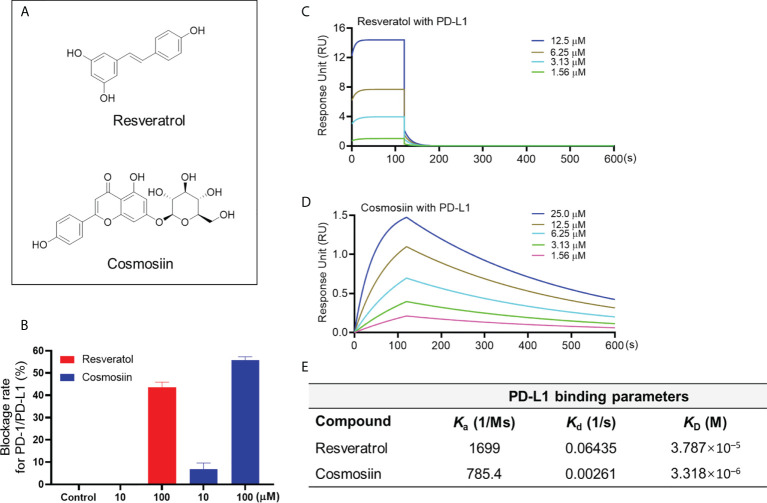
Chemical structure of resveratrol and cosmosiin **(A)**. Blockade rate of resveratrol and cosmosiin (at 10 and 100 μM) on the PD-1/PD-L1 interaction determined by the pair ELISA assay **(B)**. Binding profile of natural compounds at various concentrations with PD-L1 protein characterized by the SPR measurements. Sensograms of resveratrol with PD-L1 **(C)** protein at 1.56-12.5 μM. Sensograms of cosmosiin with PD-L1 **(D)** protein at 1.56-25 μM. Summarized binding parameters including Ka, Kd, and K_D_ between test compounds and PD-L1 proteins **(E)**.

**Figure 3:**
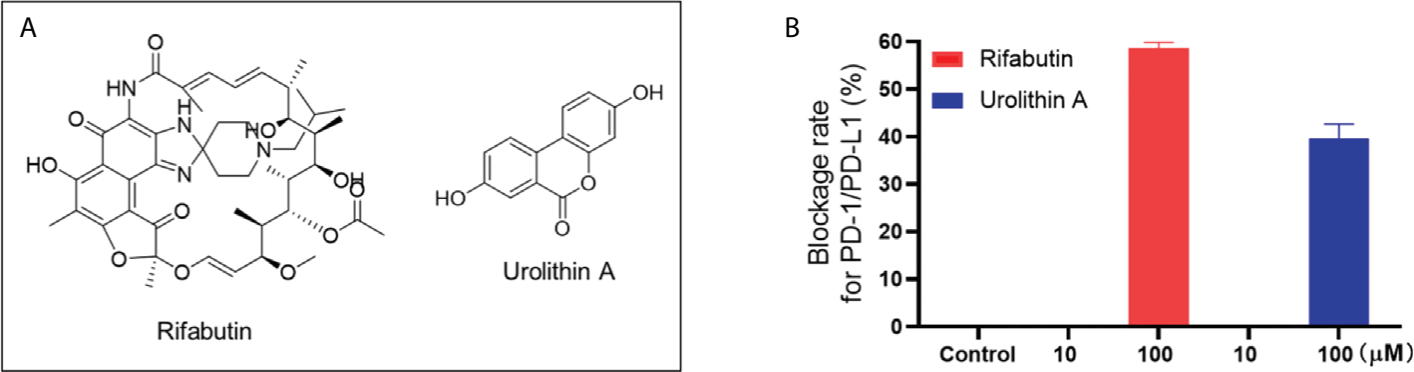
Chemical structure of rifabutin and UA **(A)**. Blockade rate of rifabutin and UA (at 10 and 100 μM) on the PD-1/PD-L1 interaction determined by the pair ELISA assay **(B)**.

This group of natural compounds included resveratrol and cosmosiin (**Figure 2A**), which showed the PD-1/PD-L1 blockade effect in the pair ELISA assay by 43.5 and 55.8%, respectively (at a concentration of 100 μM). Notably, resveratrol and cosmosiin displayed a selective binding to the PD-L1 protein with a K_D_ value of 3.79 ×10^-5^ and 3.32 ×10^-6^ M, respectively (**Figures 3C–E**). This is in agreement with a reported study using *in silico* methods (including computational docking and molecular dynamic simulation) to predict that resveratrol can facilitate the dimerization of PD-L1 (10). However, to date, resveratrol’s effect on the dimerization of PD-L1 is not confirmed by experimental data. This limitation is, at least partially, due to the lack of feasible functional assays to evaluate the dimerization of PD-L1. Therefore, specific functional assays, i.e. *PD-L1 dimerization assay*, are crucial for the development of natural compounds based PD-1/PD-L1 inhibitors. Nevertheless, apart from the dimerization of PD-L1, other mechanisms may also be involved in the disruption of PD-1/PD-L1 interaction. For instance, resveratrol was reported to disrupt the PD-1/PD-L1 interaction by altering the structure of PD-L1 protein (*via* post-translational modifications, i.e., N-linked glycosylation), accumulating abnormally glycosylated form of PD-L1, and reducing tumor cells induced cytotoxicity to T cells (10). Therefore, compounds in this category may have the scaffold of lead compounds for PD-1/PD-L1 inhibitors but further structural modifications (by medicinal chemistry) and biological evaluations (with proper functional assays) are warranted.

### Category III: PD-1/PD-L1 inhibitors without binding capacity (rifabutin and urolithin A)

Natural compounds in this category only showed a weak blockade effect on the interaction of PD-1/PD-L1. Rifabutin and urolithin A (UA; **Figure 3A**) blockaded the interaction of PD-1/PD-L1 by 58.6 and 39.7% at 100 μM, respectively (**Figure 3B**). This is in agreement with a reported study showing that rifabutin had a moderate blockade effect of PD-1/PD-L1 in a homogenous AlphaLISA assay (IC_50_ = 25 μM) (11). Similarly, a study supported the inhibitory effect of UA on PD-1/PD-L1 interaction by showing that urolithins may exert a sensitizing effect in the inhibition of immune checkpoint targeting the PD-1/PD-L1 interaction (17). However, the binding affinity between these compounds and PD-1 or PD-L1 protein was not detectable by the SPR assay. It is possible that these compounds may have shown false positive results. Further functional assays, preferably using cellular or *in vivo* models, are warranted to further evaluate the anti-PD-1/PD-L1 activity of these compounds to avoid false positive outcomes. For instance, a cellular assay, namely, the PD-1/NFAT reporter assay (18), using a recombinant Jurkat cell line, can be used as a functional assay to validate the anti-PD-1/PD-L1 effects of natural compounds.

### Category IV: PD-1/PD-L1 binders without blockade effect (pentagalloyl glucose and ellagic acid)

**Figure 4:**
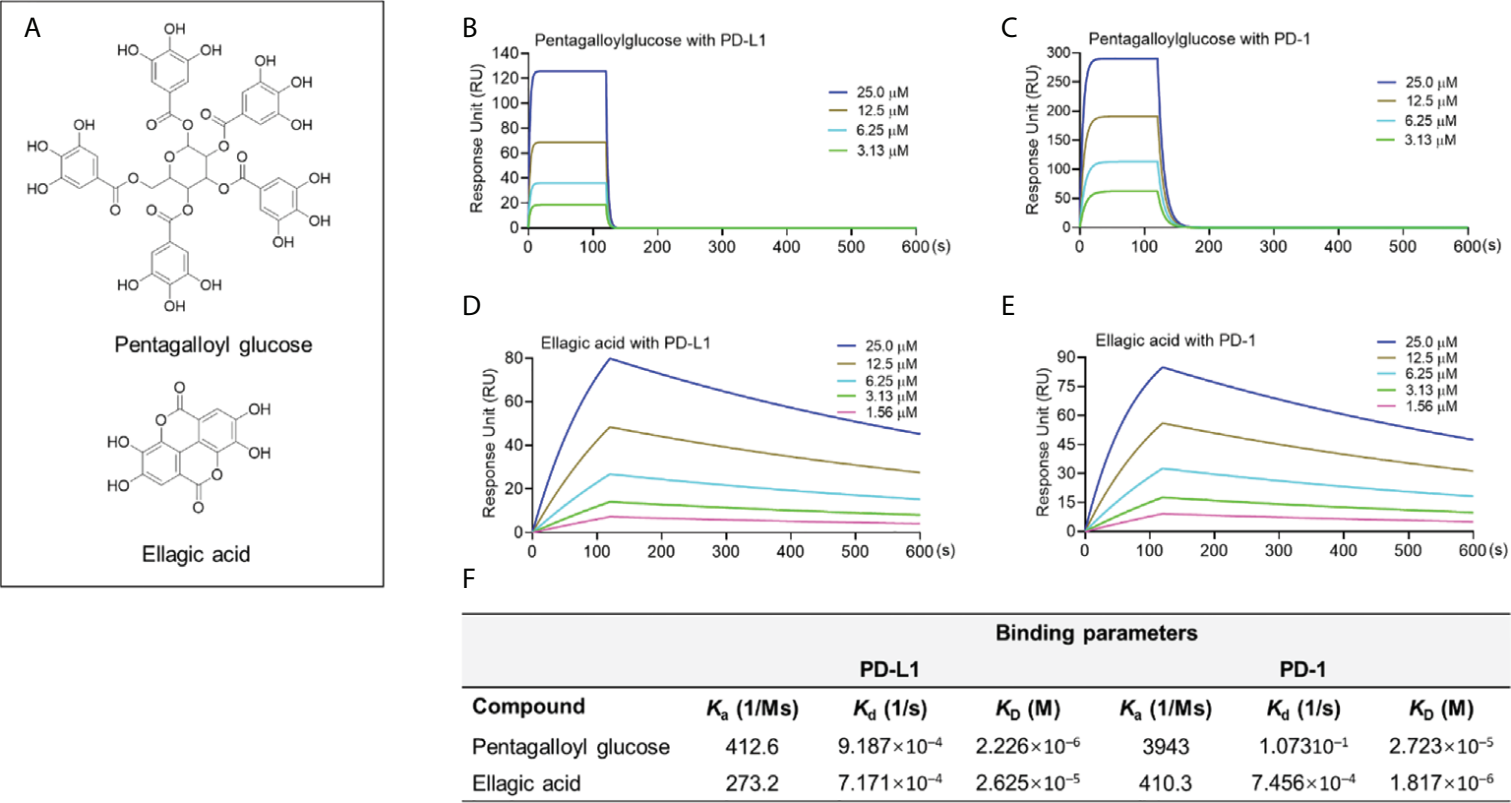
Chemical structure of PGG and EA **(A)**. Binding profile of natural compounds at various concentrations with PD-1 and PD-L1 proteins characterized by the SPR measurements. Sensograms of PGG with PD-L1 **(B)** or PD-1 protein **(C)** at 3.13-25 μM. Sensograms of EA with PD-L1 **(D)** and PD-1 **(E)** protein at 1.56-25 μM. Summarized binding parameters including Ka, Kd, and K_D_ between test compounds and PD-L1 and PD-1 proteins **(F)**.

Compounds in this group including two hydrolyzable tannins pentagalloyl glucose (PGG) and ellagic acid (EA; **Figure 4A**) were able to bind to the PD-L1 proteins. PGG and EA bound to PD-L1 protein with a K_D_ value of 2.23×10^-6^ and 2.62×10^-5^ M, respectively. PGG and EA also bound to PD-1 protein in the SPR assay with a K_D_ value of 2.72×10^-5^ and 1.82×10^-6^ M, respectively (**Figures 4B–F**). However, their blockade effect on the PD-1/PD-L1 interaction was not detected in the pair ELISA assay. To avoid possible false negative results, a PD-L1 dimerization assay should be used to confirm that they are not able to blockade the PD-1/PD-L1 interaction. The challenges shown in these assays were not surprising given that the search for small molecules targeting the PD-1/PD-L1 interface can be challenging. This is because, from a structural perspective, it requires the small molecule inhibitors to be fixed at the center of the PD-L1 homodimer (19), which is a surface with a deep hydrophobic pocket contributing to molecular interactions between the PD-L1 monomers [see **Supplementary Data Figure S1**]. Thus, although PGG and EA were able to bind to PD-L1, they may not able to be located at the desired binding pocket in the PD-L1 dimer complex to exert the blockade effect. Similar to the aforementioned limitation, a PD-L1 dimerization assay should be used to discrete the false negative results from compounds in this category. It should be noted that, apart from the PD-L1 dimerization, other small molecules may confer the anti-PD-1/PD-L1 effects *via* other mechanisms of action of PD-1/PD-L1 interaction. These compounds may also be considered as in a distinct category, which should be further investigated.

## Conclusion

A combination of a PD-1/PD-L1 blockade assay (pair ELISA) and a target-binding assay (SPR) was used to screen the PD-1/PD-L1 blockade effect of a series of natural compounds. Based on their responses from the assays, these compounds were categorized into four groups: I) PD-1/PD-L1 inhibitors bind to PD-1 and PD-L1; II) PD-1/PD-L1 inhibitors selectively bind to PD-L1 protein; III) non-binder PD-1/PD-L1 inhibitors, and IV) PD-1/PD-L1 binders without blockade effect. Further functional assays, such as the PD-L1 dimerization assay, should be used to confirm the anti-PD-1/PD-L1 activity to avoid false positive (in the I and III groups) and false negative (in the IV group) outcomes. A promising positive ‘hit’ should exhibit both potent PD-1/PD-L1 blockade activity and desirable binding affinity with the specific target proteins (i.e. PD-L1). Despite it is challenging to identify natural compounds-based PD-1/PD-L1 inhibitors, compounds in the II group may serve as lead compounds for further structural modifications to improve their PD-1/PD-L1 blockade effect. However, a larger sample size of compounds (e.g. the type II compounds) with promising blockade effect and binding capacity should be included in future studies to evaluate their biological effects in the functional assays. The current study is limited by a confined number of representing compounds included in the bioassays due to, at least partially, the fact that the discovery and development of natural products based PD-1/PD-L1 inhibitors are still in the early stage. In summary, observation demonstrated in the current study suggest that, although natural product may exert PD-L1/PD-L1 blockade effect with target protein binding capacity, their effectiveness should be verified by multiple functional bioassays to exclude false results. Further investigations using medicinal chemistry approaches are warranted to optimize the PD-1/PD-L1 blockade effects of lead natural compounds.

## Data availability statement

The original contributions presented in the study are included in the article/**Supplementary Material**. Further inquiries can be directed to the corresponding authors.

## Author contributions

HL performed the experiments. NS revised the manuscript. CL designed the methods, guided data analysis, and wrote the draft. CL and HM conceived the project and HM extensively edited the manuscript. All authors contributed to the article and approved the submitted version.

## Acknowledgments

This research was made possible in part using the Biacore T200 instrument available through the Rhode Island Institutional Development Award (IDeA) Network of Biomedical Research Excellence from the National Institute of General Medical Sciences of the National Institutes of Health under grant number P20GM103430.

## Conflict of interest

The authors declare that the research was conducted in the absence of any commercial or financial relationships that could be construed as a potential conflict of interest.

## Publisher’s note

All claims expressed in this article are solely those of the authors and do not necessarily represent those of their affiliated organizations, or those of the publisher, the editors and the reviewers. Any product that may be evaluated in this article, or claim that may be made by its manufacturer, is not guaranteed or endorsed by the publisher.

## References

[1] Chen L Han X. Anti–PD-1/PD-L1 therapy of human cancer: past, present, and future. J Clin Invest (2015) 125(9):3384–91. doi: 10.1172/JCI80011 PMC4588282 26325035

[2] Goodman A Patel SP Kurzrock R. PD-1–PD-L1 immune-checkpoint blockade in b-cell lymphomas. Nat Rev Clin Oncol (2016) 14(4):203–20. doi: 10.1038/nrclinonc.2016.168 27805626

[3] Sunshine J Taube JM. PD-1/PD-L1 inhibitors. Curr Opin Pharmacol (2015) 23:32–8. doi: 10.1016/J.COPH.2015.05.011 PMC4516625 26047524

[4] Reilly RM Sandhu J Alvarez-Diez TM Gallinger S Kirsh J Stern H. Problems of delivery of monoclonal antibodies. pharmaceutical and pharmacokinetic solutions. Clin Pharmacokinet (1995) 28(2):126–42. doi: 10.2165/00003088-199528020-00004 7736688

[5] Akinleye A Rasool Z. Immune checkpoint inhibitors of PD-L1 as cancer therapeutics. J Hematol Oncol (2019) 12(1):1–13. doi: 10.1186/S13045-019-0779-5 PMC6729004 31488176

[6] Zhan MM Hu XQ Liu XX Ruan BF Xu J Liao C. From monoclonal antibodies to small molecules: the development of inhibitors targeting the PD-1/PD-L1 pathway. Drug Discovery Today (2016) 21(6):1027–36. doi: 10.1016/J.DRUDIS.2016.04.011 27094104

[7] Ganesan A Ahmed M Okoye I Arutyunova E Babu D Turnbull WL. Comprehensive *in vitro* characterization of PD-L1 small molecule inhibitors. Sci Rep (2019) 9(1):1–13. doi: 10.1038/S41598-019-48826-6 PMC6712002 31455818

[8] Kim JH Kim YS Choi JG Li W Lee EJ Park JW. Kaempferol and its glycoside, kaempferol 7-o-rhamnoside, inhibit PD-1/PD-L1 interaction *In vitro* . Int J Mol Sci (2020) 21(9):3239. doi: 10.3390/IJMS21093239 PMC7247329 32375257

[9] Choi JG Kim YS Kim JH Kim TI Li W Oh TW. Anticancer effect of salvia plebeia and its active compound by improving T-cell activity *via* blockade of PD-1/PD-L1 interaction in humanized PD-1 mouse model. Front Immunol (2020) 11:598556/FULL. doi: 10.3389/FIMMU.2020.598556/FULL 33224152 PMC7674495

[10] Verdura S Cuyàs E Cortada E Brunet J Lopez-Bonet E Martin-Castillo B. Resveratrol targets PD-L1 glycosylation and dimerization to enhance antitumor T-cell immunity. Aging (Albany NY) (2020) 12(1):8. doi: 10.18632/AGING.102646 31901900 PMC6977679

[11] Patil SP Yoon SC Aradhya AG Hofer J Fink MA Enley ES. Macrocyclic compounds from ansamycin antibiotic class as inhibitors of PD1–PDL1 protein–protein interaction. Chem Pharm Bull (2018) 66(8):773–8. doi: 10.1248/CPB.C17-00800 30068796

[12] Liu C Seeram NP Ma H. Small molecule inhibitors against PD-1/PD-L1 immune checkpoints and current methodologies for their development: a review. Cancer Cell Int (2021) 21(1):1–17. doi: 10.1186/S12935-021-01946-4 33906641 PMC8077906

[13] Yuan T Ma H Liu W Niesen DB Shah N Crews R. Pomegranate’s neuroprotective effects against alzheimer’s disease are mediated by urolithins, its ellagitannin-gut microbial derived metabolites. ACS Chem Neurosci (2016) 7(1):26–33. doi: 10.1021/ACSCHEMNEURO.5B00260 26559394

[14] Ma H Liu W Frost L Wang L Kong L Dain JA. The hydrolyzable gallotannin, penta- O -galloyl-β- d -glucopyranoside, inhibits the formation of advanced glycation endproducts by protecting protein structure. Mol Biosyst (2015) 11(5):1338–47. doi: 10.1039/C4MB00722K 25789915

[15] Zak KM Grudnik P Guzik K Zieba BJ Musielak B Dömling A. Structural basis for small molecule targeting of the programmed death ligand 1 (PD-L1). Oncotarget (2016) 7(21):30323. doi: 10.18632/oncotarget.8730 27083005 PMC5058683

[16] Wang H Oo Khor T Shu L Su ZY Fuentes F Lee JH. Plants against cancer: A review on natural phytochemicals in preventing and treating cancers and their druggability. Anti-cancer Agents medicinal Chem (2012) 12(10):1281. doi: 10.2174/187152012803833026 PMC4017674 22583408

[17] Zitvogel L Kroemer G. Boosting the immunotherapy response by nutritional interventions. J Clin Invest (2022) 132(11):e161483. doi: 10.1172/JCI161483 35642631 PMC9151683

[18] Wang X Martin AD Negri KR McElvain ME Oh J Wu ML. Extensive functional comparisons between chimeric antigen receptors and T cell receptors highlight fundamental similarities. Mol Immunol (2021) 138:137–49. doi: 10.1016/J.MOLIMM.2021.07.018 34419823

[19] Guzik K Zak KM Grudnik P Magiera K Musielak B Torner R. Small-molecule inhibitors of the programmed cell death-1/Programmed death-ligand 1 (PD-1/PD-L1) interaction *via* transiently induced protein states and dimerization of PD-L1. J Medicinal Chem (2017) 60(13):5857–67. doi: 10.1021/ACS.JMEDCHEM.7B00293 28613862

