# Supplementary material for: Further investigation of blockade effects and binding affinities of selected natural compounds to immune checkpoint PD-1/PD-L1
Source: Front Oncol. 2022 Sep 12;12:995461. doi: 10.3389/fonc.2022.995461 (PMC9511049; doi:10.3389/fonc.2022.995461)
Supplement: Supplementary file 1 [file Table_1.docx]

**Supplementary Material**

**Further Investigation of Blockade Effects and Binding Affinities of Selected Natural Compounds to Immune Checkpoint PD-1/PD-L1**

Huifang Li, Navindra P. Seeram, Chang Liu*, Hang Ma*

Bioactive Botanical Research Laboratory, Department of Biomedical and Pharmaceutical Sciences, College of Pharmacy, University of Rhode Island, Kingston, Rhode Island, USA

*** Correspondence:**Chang Liu
hichang813@uri.edu

Hang Ma

hang_ma@uri.edu

**Figure S1**.


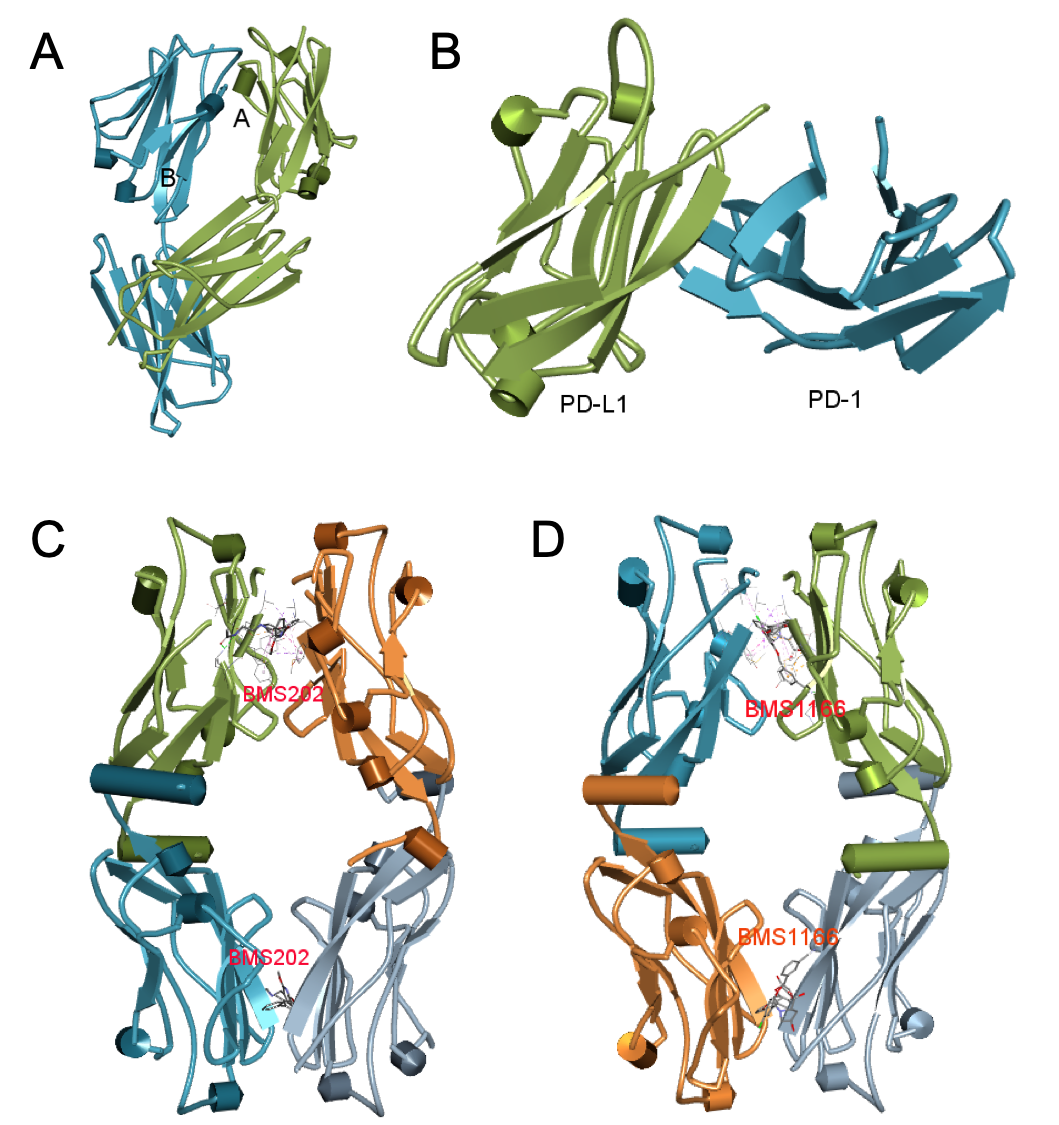


**Figure S1**. (A) The structure of human PD-L1(4Z18) and the structures of human PD-L1 and PD-l (4ZQK). The structure of PD-L1 with inhibitor BMS202 (C) and BMS1166 (D) demonstrated that the presence of BMS 202 or BMS1166 facilitates the dimerization of PD-L1.
